# HIV-1 bispecific antibody iMab-N6 exhibits enhanced breadth but not potency over its parental antibodies iMab and N6

**DOI:** 10.1186/s12985-022-01876-1

**Published:** 2022-09-07

**Authors:** Tumelo Moshoette, Maria Antonia Papathanasopoulos, Mark Andrew Killick

**Affiliations:** HIV Pathogenesis Research Unit, Department of Molecular Medicine and Haematology, Faculty of Health Sciences, University of the Witwatersrand, 7 York Road, Parktown, Johannesburg, 2193 South Africa

**Keywords:** HIV-1 prevention, HIV-1 therapy, Broadly neutralizing antibodies, Bispecific antibodies, Bliss-Hill potency prediction

## Abstract

The recently published AMP trial (HVTN 703/HPTN 081 and HVTN704/HPTN 085) results have validated broad neutralising antibodies (bNAbs) as potential anti-HIV-1 agents. However, single bNAb preparations are unlikely to cope with the onslaught of existing and de novo resistance mutations, thus necessitating the use of bNAb combinations to achieve clinically relevant results. Specifically engineered antibodies incorporating two bNAbs into a single antibody structure have been developed. These bispecific antibodies (bibNAbs) retain the benefits of bNAb combinations, whilst several conformations exhibit improved neutralisation potency over the parental bNAbs. Here we report on the engineering of a bibNAb comprising of an HIV-1 spike targeting bNAb N6 and a host CD4 targeting antibody ibalizumab (iMab). Antibodies were expressed in HEK293T cells and purified by protein-A affinity chromatography followed by size exclusion chromatography to achieve homogenous, monomeric, bibNAb preparations. Antibody purity was confirmed by SDS-PAGE whilst epitope specificity and binding were confirmed by ELISA. Finally, antibody breadth and potency data were generated by HIV-1 neutralisation assay (n = 21, inclusive of the global panel). iMab-N6 exhibited better neutralisation breadth (100% coverage) in comparison to its parental bNAbs iMab (90%) and N6 (95%). This is encouraging as exceptional neutralisation breadth is necessary for HIV-1 treatment or prevention. Unfortunately, iMab-N6 did not exhibit any enhancement in potency over the most potent parental antibody, iMab (*p* = 0.1674, median IC_50_ of 0.0475 µg/ml, and 0.0665 µg/ml respectively) or the parental combination, iMab + N6 (*p* = 0.1964, median IC_50_: combination 0.0457 µg/ml). This result may point to a lack of dual engagement of the bibNAb Fab moieties necessary for potency enhancement. Against the previously reported bibNAbs; iMab-CAP256, 10E08-iMab, and PG9-iMab; iMab-N6 was the lowest performing bibNAb. The re-engineering of iMab-N6 to enhance its potency, while retaining breadth, is a worthwhile endeavour due to its clinical potential.

**Supplementary Information:**

The online version contains supplementary material available at 10.1186/s12985-022-01876-1.

## Introduction

Broadly neutralising antibodies (bNAbs) isolated from a subset of HIV-1 positive individuals are ideal candidates for development as novel anti-HIV-1 agents [1]. These antibodies neutralise the virus through targeting highly conserved regions on the HIV-1 spike, namely, the V2 apex, V3 glycan, CD4 binding site (CD4bs), the silent face, interface-FP, and the membrane-proximal external region [2]. It is the targeting of these conserved regions that gives bNAbs improved breadth and potency over HIV-1 neutralising antibodies (NAbs) [3]. The poor neutralization coverage and lower potency of first generation monoclonal neutralizing antibodies (Nabs) [4] (e.g., b12, 447-52D, 2G12, 2F5 and 4E10) against a range of clinically relevant HIV-1 cast doubt on the clinical use of these NAbs as anti-HIV-1 agents. However, the isolation of more contemporary bNAbs (e.g., CAP256-VRC26.25 [5], 10E8 [6], N6 [7], 1–18 [8], VRC01 [9], and 3BNC117 [10] and the N49 antibody lineage [11] to name a few) has reinvigorated the field as many potential clinical candidates have been identified [12]. Generally, CD4bs bNAbs (e.g., N6 [7], VRC07-523 [13], 1–18 [8], and 3BNC117 [10]) offer the best combination of breadth and potency whereas V2 antibodies (e.g., CAP256-VRC26.25 [5], PGDM1400 [14]) offer superior potency coupled with moderate breadth [2].

Broadly neutralising antibodies are currently being investigated for use as HIV-1 pre-exposure prophylaxis (PrEP) and/or therapy. As PrEP, bNAbs such as CAP256-VRC26.25 and PGDM1400 [15], PGT121 [16], and VRC01 [17] were shown to protect non-human primates (NHP) following SHIV challenges. Encouragingly, the highly potent V2 specific bNAbs; PGDM1400 and CAP256-VRC26.25 conferred protection against subtype C SHIV-325c with a very low serum concentration of 2.5 and < 0.75 µg/ml, respectively; consistent with the extraordinary potency of these antibodies in vitro [15]. However, these researchers observed breakthrough infections as a result of variability in the V1/V2 loop sequence of the SHIV viral challenge stock that was able to convey resistance against the PGDM1400 antibody [15]. Indeed, the existence and/or emergence of HIV-1 resistance mutations is a major concern in single bNAb PrEP intervention strategies, as no single bNAb has demonstrated 100% neutralization coverage of clinically relevant, HIV-1 Env-pseudotyped panels in vitro. This limitation was recently accentuated in a major phase IIb clinical trial (HVTN 703/HPTN 081 and HVTN704/HPTN 085; AMP) where VRC01 (CD4bs antibody) offered no protection against HIV-1 acquisition compared to the placebo control group (saline infusion) [18]. Closer inspection of the data revealed that only 30% of the circulating viruses within these trial participants were sensitive to VRC01 (IC_80_ < 1 µg/ml), thereby rendering the bNAb ineffective against the majority of the circulating viruses [comprising of intermediate (IC_80_ = 1–3 µg/ml) and resistant (IC_80_ > 3 µg/ml) viruses] [18]. Against the sensitive viruses, VRC01 was 75% effective at preventing HIV-1 acquisition, failing in the other 25% due to the emergence of new resistance mutations [18]. This trial underscores the importance of bNAb breadth and potency, and choosing the correct bNAb, in offering full protection against HIV-1 acquisition within the real world, clinical setting. As disappointing as these results are, the AMP trial has validated bNAbs as potential PrEP candidates subject to a selection that maximises neutralising breadth and potency of the circulating regional/global HIV-1 strains.

As therapeutic agents, bNAbs such as PGT121 and 3BNC117 [19], N6-LS [20], and 1–18 [8] have been shown to suppress HIV-1 viral load in either humanised mice or NHP. However, viral rebound was detected due to the emergence of resistance mutations against 3BNC117 and as a result of declining N6-LS and PGT121 plasma levels [19, 20]. Interesting, 1–18 fully suppressed HIV-1 in humanised mice without selecting for resistance mutations, indicating a high genetic barrier to resistance [8]. BNAb therapy has also been investigated in a number of small clinical trials where 3BNC117 (phase 1: NCT02018510 and phase IIa: NCT02446847), VRC01 (phase I: NCT01950325 and NCT02463227), 10-1074 (phase I: NCT02511990) and PGT121 (NCT02960581) were shown to transiently suppress viral load in viraemic individuals [21–24] or prevent viral rebound during treatment interruption [25, 26]. However, pre-existing resistant viruses coupled with the emergence of new resistant viruses rendered these treatments ineffective overtime [21–23, 25, 26]. As with bNAb PrEP, these results once again underscore the importance of antibody breadth.

To improve neutralization coverage, appropriate bNAb combinations with proven synergistic effects may be used in place of bNAb monotherapy. Modelling, in vitro and in vivo studies have shown that bNAb combinations offer improved neutralisation coverage and potency over bNAb monotherapy [27–30]. Moreover, in a phase Ib clinical trial (NCT02825797), bNAb combination (3BNC117 and 10-1074) was shown to be more effective than bNAb monotherapy (3BNC117) at preventing viral rebound following treatment interruption (median viral rebound of 21 weeks and 6–10 weeks respectively) [31]. In a separate phase Ib clinical trial (NCT02825797), the 3BNC117 and 10-1074 combination was shown to supress viral load in viraemic individuals for a significantly longer period than either 3BNC117 or 10-1074 monotherapy [21, 22, 32]. However, in both studies, differences in bNAb half-life (3BNC117: 17.6 or 12.3 days, respectively, and 10-1074: 23.2 or 12.7 days, respectively) meant 3BNC117 levels dropped faster than 10-1074 effectively causing monotherapy; this in turn resulted in viral rebound driven by 10-1074 resistance [31, 32]. These differences in bNAb pharmacokinetics and the cost implications of bNAb combination PrEP/therapy are what makes this strategy challenging to implement in mainstream clinical settings.

Specifically engineered antibodies incorporating two or three bNAbs into one antibody structure have been developed as possible replacements for bNAb combinations. These bi/tri-specific antibodies (bi/tribNAbs) retain the benefits of bNAb combinations without any of the associated challenges. Several bi/tribNAbs reported in the literature (10E8v4-iMab [33], PG9-iMab [34], iMab-CAP256 [35], VRC01/PGDM1400-10E8v4 [36], N6/PGDM1400-10E8v4 [36], and 10E08/Bi-ScFv_dVRC01-5X-PGT121_ [37]) exhibit greater breadth and potency than their parental bNAb combination. This enhancement in potency is due to the simultaneous binding of the paratopes resulting in increased avidity. For the iMab-based bibNAbs, the localisation/concentration of the bibNAbs at the site of entry (surface of the CD4 T-cells) positively contributes to the potency enhancement [34]. IMab, a humanised mouse antibody exhibits broad-spectrum neutralization of HIV-1 viral isolates through targeting a conformational epitope on the second N-terminal, extracellular domain of the human CD4 receptor [38, 39]. iMab does not compete for HIV-1 gp120 envelope (Env) binding to the CD4+ T cell receptor, but rather inhibits viral infection through sterically hindering the post-binding rearrangement steps required for viral and host target cell membrane fusion [39, 40]. Importantly, iMab displays an excellent safety profile in vivo as its binding does not interfere with CD4-mediated major histocompatibility complex II immune signalling functions [41, 42]. Combined, these properties make iMab a suitable candidate for generating bispecific antibodies in combination with HIV-1 Env targeting bNAbs. Clinical trials are currently underway to evaluate the safety, tolerability, pharmacokinetics, and anti-viral activity of bibNAb 10E8v4-iMab (NCT03875209) and tribNAb VRC01/PGDM1400-10E8v4 (NCT03705169).

Although promising, the prohibitive cost associated with antibody therapy limits their large-scale implementation compared to more traditional ART-based strategies. Alternative plant-based expression systems for the expression of bNAbs are being investigated to reduce the associated manufacturing costs [43]. Moreover, antibody engineering strategies (LS-mutations) to improve serum half-lives and reduce the frequency of antibody administration [15, 20]; and vector-mediated immunoprophylaxis strategies that result in sustained antibody expression in vivo and protection against HIV-1 acquisition in preclinical trials, are also being investigated as cost reduction strategies [44–46]. These strategies may be combined to further reduce the cost of antibody-based PrEP or therapy against HIV-1 and allow for their clinical implementation. Whatever strategy is employed, antibody therapy is promising.

We previously reported on a highly potent and broad bibNAb iMab-CAP256 [35]; specifically engineered with HIV-1 subtype C in mind. HIV-1 subtype C is the dominant subtype in Southern Africa and by extension the world [47]. Overcoming the HIV-1 pandemic therefore requires PrEP or therapeutic agents that encompass this subtype. Here we report on the engineering and characterisation of an additional bibNAb comprising of an HIV-1 Env targeting bNAb N6 [7] and the host CD4 targeting humanised monoclonal antibody ibalizumab (iMab) [38–40]. Huang et al. reported on N6’s superior performance against a panel of 181 pseudoviruses, achieving a neutralisation coverage of 98% with a median IC_50_ of 0.038 µg/ml [7]. Crucially, N6 was shown to have an exceptional neutralisation coverage of 98% with a median IC_50_ of 0.066 µg/ml against HIV-1 subtype C [7]. Moreover, N6 was the best performing out of 8 bNAbs against HIV-1 subtype C with a 95–96% IC_80_ coverage [30]. Combined, these data suggest that the incorporation of N6 into combination-based therapies or inclusion into bi/tribNAb configurations would be beneficial. The monoclonal antibody iMab has a neutralisation coverage of 92% against diverse HIV-1 Env subtypes with a median IC_50_ of 0.03 µg/ml [48]. iMab (Trogarzo®) is the only FDA approved monoclonal antibody for use as HIV-1 salvage therapy [49]. By combining N6 with iMab, we hope to produce a bibNAb with exceptional breadth and enhanced potency especially against HIV-1 subtype C. To this end, we describe the engineering of iMab-N6 bibNAb, confirm the functionality of individual Fab binding regions in the bispecific antibody configuration and demonstrate an improved breadth relative to the parental bNAbs against a panel of 21 diverse HIV-1 pseudoviruses.

## Methodology

### Reagents used in this study

The single plasmid, mAb expression vector (pMin) was generously donated by Balazs [44, 45] and modified to remove the CMV promoter and ITR flanking regions for optimal in vitro antibody expression. The following reagents were obtained from the NIH AIDS Research and Reference Reagent Program, Division of AIDS, NIAID (contributor in parentheses): N6 antibody heavy and light chain expression vectors (Drs. Jinghe Huang and Mark Connors [7]). Reagents for HIV-1 pseudovirus production including: TZM-bl (JC53-bl) reporter cell line (Drs. John C. Kappes, Xiaoyun Wu and Transzyme Inc. [50–54]), pSG3Δenv (Drs. John C. Kappes and Xiaoyun Wu [54, 55]) and 21 complementing HIV-1 Env plasmids 25710, 246-F3.C10.2, 398f1 and CH119 (Drs. C. Williamson, M. Hoelscher, L. Maboko, and D. Montefiori [56, 57]), CNE8 and CNE55 (Drs. L. Zhang, H. Shang, and D. Montefiori [56, 58]), PVO.4 and QH0692.42 (Drs. D. Montefiori, F. Gao and M. Li [59]), CAP210.2.00.E8 and CAP45.2.00.G3 (Drs. L. Morris, K. Mlisana and D. Montefiori [60]), DU156.12 (Drs. D. Montefiori F. Gao, S. Abdool Karim and G. Ramjee [60, 61]), DU422.01 (Drs. D. Montefiori, F. Gao, C. Williamson and S. Abdool Karim [60, 61]), ZM53 M.PB12 and ZM135 M.PL10a (Drs. E. Hunter and C. Derdeyn [60]), X1632 (Dr. D. Montefiori [56, 62]), TRO11 (Drs. F. Gao and D. Montefiori [56, 59]), X2278 and BJOX2000 (Drs. M. Thomson, A. Revilla, E. Delgado, and D. Montefiori [56]), CE0217 and CE1176 (Drs. R. Swanstrom, L. Ping, J. Anderson, and D. Montefiori [56]), RHPA4259 (Drs. B. H. Hahn and Dr. J. F. Salazar-Gonzalez [59]). The HIV-1 global pseudovirus panel (Cat #12670) [56] is included in the abovementioned 21 plasmids.

### Antibody construct design and synthesis

**Fig. 1:**
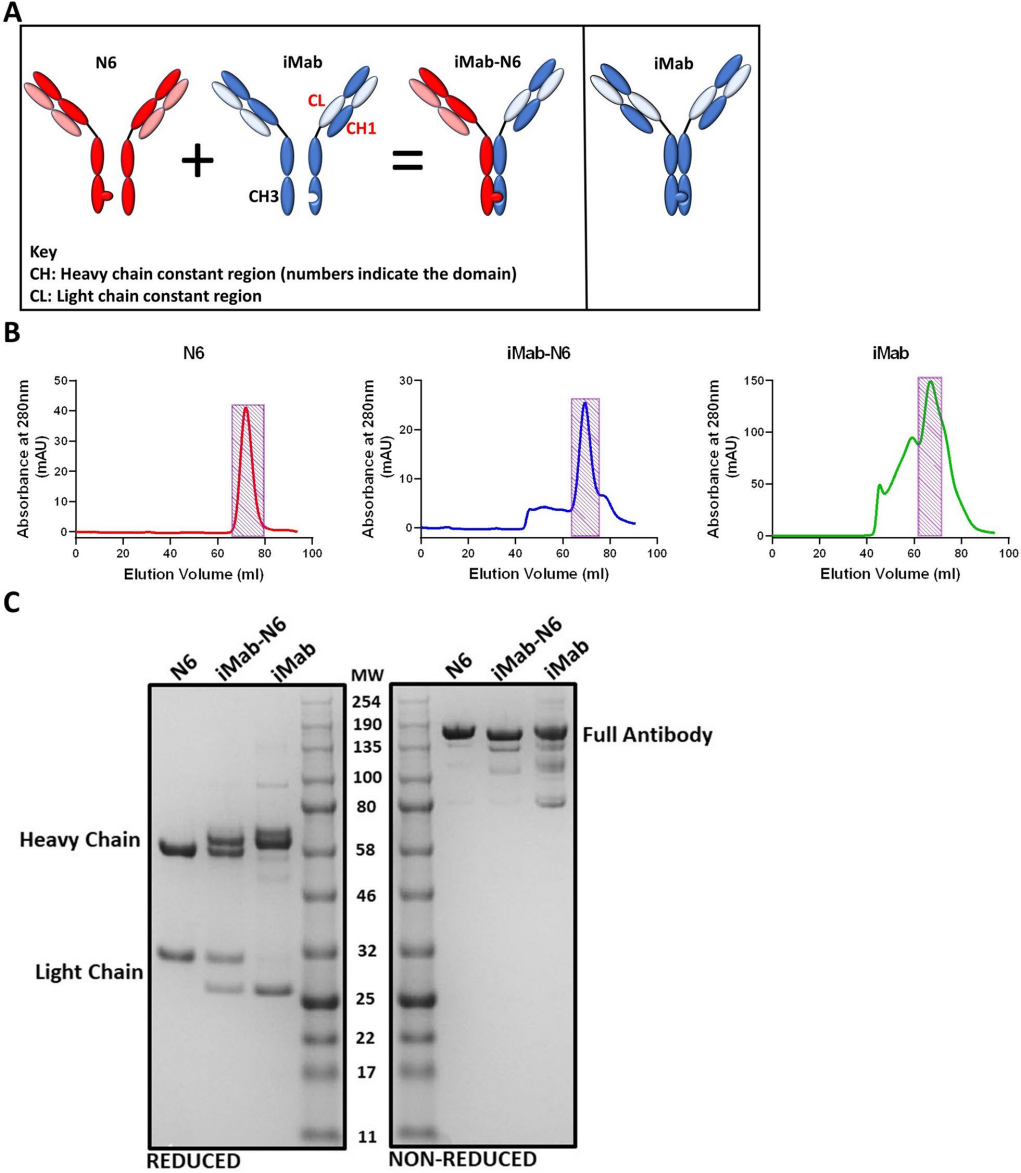
Bispecific antibody design, purification and SDS-PAGE analysis. **A** Left panel: Schematic of iMab-N6 engineered using knob-in-a-hole mutations in the CH3 region of N6 and CrossMAb^CH1_CL^ mutations in iMab. These mutations promote preferential assembly of iMab-N6 by limiting the formation of by-products. **A** Right panel: To show that these mutations do not affect the function of the antibody, iMab monoclonal antibody was engineered with knob-in-a-hole and CrossMAb^CH1_CL^ mutations. **B** Chromatograms of size exclusion chromatography (SEC) purified antibodies with elution fractions corresponding to the correctly assembled antibody confirmation represented by the shaded rectangles. **C** Antibodies were resolved on a gradient SDS-PAGE post SEC under reduced and non-reduced conditions

The bispecific antibody expression constructs were engineered as described by Moshoette et al. [35]. Briefly, iMab-N6 was designed in silico using SnapGene by introducing the fragment antigen-binding region (Fab fragment) sequences of iMab [40] and N6 [33] (PDB: 3O2D and 5TE7 respectively) to their respective bibNAb constructs. Additionally, Knob-in-a-hole [63] (KiH) and CrossMab^CH1_CL^ [64] mutations were introduced into the sequences to optimise the correct assembly of the bibNAb (Fig. 1a). CrossMab^CH1_CL^ and “hole” (L368A, Y349C and Y407V) mutations were introduced into the host targeting iMab whilst the corresponding “knob” (T366W and S254C) mutations were incorporated into the HIV-1 Env targeting N6 to create a host-Env dual targeting bibNAb (Fig. 1A). Parental N6 antibody heavy and light chain constructs were sourced from the NIH AIDS Research and Reference Reagent Program (ARP-12966 and ARP-12967, respectively). iMab was engineered using the same bibNAb modifications (CrossMab^CH1−CL^ and KiH) to serve as an assembly control (Fig. 1A, rightmost panel). The Ab designs were sent to GeneArt (ThermoFisher Scientific, Waltham, MA, USA) for codon optimisation and construct synthesis. The synthesised antibody gene constructs were subcloned into a mammalian expression vector provided courtesy of Balazs. Large scale plasmid production was performed in *E. coli* DH5α cells and purified using the Qiagen Maxi plasmid isolation kit (Qiagen, Hilden Germany) according to the manufacturer’s protocol.

### Antibody expression and purification

Antibody production was performed as described previously [35, 65]. Briefly, 24 h before transfection, HEK293T cells were seeded in a tissue culture flask and allowed to grow to 70% confluency. After 24 h, transient transfection was done using a 1:3 DNA to PEI MAX (Polysciences Inc., PA, USA) ratio and the cells were incubated for a further 18 h. The cell culture media was then replaced with SFMII media (Thermo Fisher Scientific, Waltham, MA, USA) supplemented with 2 mM glutamax (Thermo Fisher Scientific, Waltham, MA. USA). The transfected cells produced and secreted the antibodies into the cell culture supernatant which was harvested every 2 days up to a maximum of 10 days. The harvested supernatants were filtered through 0.2 µm filters and stored at 4 °C until further processing. Antibody purification was done by means of Protein-A affinity chromatography (Sigma-Aldrich, St Lois, M0, USA) followed by Size Exclusion Chromatography (SEC) on a Superdex 200 PG 16/600 HiLoad column (GE Healthcare, Chicago, IL, USA) to attain a pure sample comprising of monomer antibodies. SEC fractions containing the pure Ab sample (as shown by the chromatograms) were pooled, filtered with a 0.2 µm filter and concentrated using an Amicon centrifugal-filtration device (Merck-Millipore, Burlington, MA, USA) with a molecular weight cut-off 50 kDa. The purified proteins were resolved on a NuPAGE™ Bis–Tris Mini Gel (Polyacrylamide percentage: 8%, 10%, 12%, and 4–12%, Thermo Fisher, Waltham, MA, USA) to confirm their purity.

### gp140_FVC_GCN4 and 4dCD4 protein production and purification

Constructs for the recombinant expression of HIV-1 gp140_FVC_GCN4 trimer or human, wildtype four domain CD4 (4dCD4) were available in our laboratory. The HIV-1 gp140_FVC_GCN4 trimer comprises an inferred Founder virus consensus C sequence (FVC), and was recombinantly expressed by stably transfected 293F cell lines and purified by a combination of lectin-affinity chromatography and SEC, to produce a functional, conformationally intact protein as described previously [66].

The 4dCD4 plasmid construct, codon-optimised for mammalian expression, was designed and obtained from GeneArt (Thermo Fisher Scientific, Waltham, MA. USA) and included an N-terminal, membrane-localisation signal that in the absence of the CD4 transmembrane and cytoplasmic regions, allows for efficient secretion of the expressed proteins. The 4dCD4 coding sequence was PCR amplified using the Q5 High-fidelity 2× Master mix (New England Biolabs, Ipswich, MA, USA) and sub-cloned into pCINeo (Promega, Madison, WI, USA) using the *Xba*I and *Not*I restriction sites. A C-terminal HIS tag (6× histine residues) was included in frame with the 4dCD4 coding sequence for downstream purification using immobilised metal affinity chromatography (IMAC). Large scale plasmid isolation was performed as described above. 4dCD4 was expressed by standard mammalian, cationic lipid transfection protocols in the FreeStyle 293F cell line (Thermo Fischer, Waltham, MA, USA). Briefly, 293F cells were maintained between 0.5 and 3.0 × 10^6^ cells/ml in FreeStyle 293 Expression medium (Thermo Fisher, Waltham, MA, USA) at 37 °C, in atmosphere supplemented with 5–8% CO_2_ on an orbital shaking platform (130 rpm). Cells were transfected at density of 1.5–2.0 × 10^6^ cell/ml, with a viability > 90% using 1 µg plasmid DNA per ml of culture. DNA:PEI MAX ratios were maintained at 1:3 ratio, and the total transfection reagent volume (including the plasmid DNA, PEI MAX, and Freestyle media) was 10% of the total culture volume. Culture supernatants containing the expressed 4dCD4 protein were harvested every second day and clarified by centrifugation (300×g, 5 min) for a maximum of 7 days. Cultures were re-seeded at 1.5 × 10^6^ cell/ml following the harvesting of the supernatants. IMAC purification of the 4dCD4 was performed using 1 ml of packed, Ni-charged resin incubated per ~ 500 ml volume of culture supernatant overnight at 4 °C with stirring to maintain the resin in suspension. The resin was collected by centrifugation at 1000×*g* for 5 min and washed with wash buffer (25 mM KH_2_PO_4_, 150 mM KCl, pH 8.0) to disrupt any nonspecific protein-resin interactions. The 4dCD4 was eluted from the resin using the wash buffer containing 250 mM imidazole, concentrated and buffer-exchanged into Dulbecco’s PBS (without calcium and magnesium) (Thermo Fisher Scientific, Waltham, MA, USA) using an Amicon centrifugation concentrating device (MW cut off 30 kDa, Merck-Millipore, Burlington, MA, USA). Concentrated protein was quantified using the BCA protein assay (Thermo Fisher Scientific, Waltham, MA, USA), and flash frozen in liquid nitrogen for storage at − 80 °C.

### Functional evaluation of bibNAb Fab moieties

To confirm the purified antibodies bind to their respective epitopes, on the recombinant HIV-1 Env gp140_FVC_GCN4 and 4dCD4, an ELISA was performed. N6 and the HIV-1 targeting arm of iMab-N6 were tested against HIV-1 Env gp140_FVC_GCN4 whereas iMab and the host targeting arm of iMab-N6 were tested against 4dCD4. In preparation for the Env ELISA, 96 well Nunc Maxisorb ELISA plates (Fisher Scientific, Waltham, MA, USA) were coated with 100 ng/well *G. nivalis* lectin (Sigma-Aldrich, St Lois, MO, USA) and incubated overnight at 4 °C. For the CD4 ELISA, serial dilutions of 4dCD4 (starting concentration of 1000 ng/ml per well; 3× dilution series) were added to an uncoated 96 well Nunc Maxisorb ELISA plate (Fisher Scientific, Waltham, MA, USA) and likewise incubated overnight at 4 °C. The plates were then blocked for an hour at room temperature (Blocking buffer: Dulbecco’s phosphate buffered saline (DPBS) (Sigma), 0.05% (v/v) Tween 20 and 1% BSA). Serial dilutions of gp140_FVC_GCN4 (starting concentration of 1000 ng/ml per well; 3× dilution series) were added to the coated Env ELISA plates followed by an hour incubation at room temperature. With plates ready, N6 was added at a uniform concentration of 0.8 µg/ml and iMab-N6 was added at a uniform concentration of either 0.8 µg/ml or 1.6 µg/ml for the Env ELISA. Both iMab and iMab-N6 were tested at a single uniform concentration of 1 µg/ml for the CD4 ELISA. Bound antibodies were detected using anti-human, horseradish peroxidase linked secondary antibody (GE Healthcare, Chicago, IL, USA) and standard chromogenic methodologies. To avoid contamination by non-bound proteins (i.e., HIV-1 Env or 4dCD4 or 1° antibodies or 2° antibodies), the plate was washed after each step with wash buffer [DPBS (Sigma) containing 0.05% (v/v) Tween 20]. Both iMab and N6 parental antibodies served as appropriate negative controls in the gp140_FVC_GCN4 and 4dCD4 ELISAs, respectively. All samples were run in duplicate.

### HIV-1 in vitro neutralisation assay

The breadth and potencies of iMab-N6, parental bNAbs (iMab or N6) and parental bNAb combination (iMab + N6) were determined against a panel of 21 geographically diverse pseudoviruses. This was done using a single round HIV-1 pseudovirus infectivity assay in the TZM-bl reporter cell line, as described previously [65]. Briefly, single-round HIV-1 Env pseudoviruses were produced by co-transfection of HEK293T cells with HIV-1 *rev/env* expression plasmids (Reagents—HIV-1 Env plasmids) and the *env*-deficient HIV-1 back bone plasmid (pSG3ΔEnv) at 1:2 ratio using FuGene HD transfection reagent (Promega, Madison, WI, USA). Culture supernatant containing the pseudoviruses was harvested at 48 h post transfection, filtered through a 0.45 µm Acrodisc filter (Pall Corporation, New York, USA), supplemented to a final concentration of 20% foetal calf serum (Gibco, Thermo Scientific) before being aliquoted into 1 ml volumes and stored at − 80 °C. Tissue culture infectious dose to achieve 50% infection (TCID_50_) was determined in the TZM-Bl cell line as described previously [59]. Neutralization assays were performed by preparing serial dilutions of the antibodies (iMab, N6, and iMab-N6) in a 96 well cell culture plate using a starting concentration of 4 µg/ml and a 5× dilution series. The parental combination (iMab + N6) was prepared using a 50/50 split of each parental Ab to a final starting concentration of 4 µg/ml and a 5× dilution series. Following Ab preparations, 200 TCID_50_ of pseudovirus was added to each well and incubated for an hour at 37 °C in 5% CO_2_. Following this, 1 × 10^4^ cells/well of freshly trypsinised TZM-bl cells were added, and the plate was incubated for a further 48 h at 37 °C in 5% CO_2_. The TZM-bl cells were prepared in complete DMEM supplemented with DEAE Dextran (Sigma-Aldrich, St Lois, Mo, USA) to a final assay concentration of 20 µg/ml; this was done to enhance the infectivity of the pseudoviruses to the TZM-bl cells. Luciferase expression was determined using Bright Glo Luciferase reagent (Promega, Madison, WI, USA) according to the manufacturer’s instructions and quantified using the Promega Glomax Explorer luminometer (Promega, Madison, WI, USA). Cell only and virus only control wells were included as appropriate controls. Viral inhibition was determined by the reduction in relative luminescence units (RLU) compared to the virus only containing wells after subtracting the background RLU values of the cells only containing wells. IC_50_ and IC_80_ values were calculated using the non-linear regression function (log (agonist) vs. response—Find ECanything) in GraphPad Prism 7 and represent the Ab concentration (μg/ml) required to achieve 50% and 80% pseudovirus inhibition respectively. iMab-CAP256, 10E8-iMab and PG9-iMab IC_50_ data was sourced from Moshoette et al. [35] for comparison to iMab-N6. Only those pseudoviruses sensitive to all parental antibody combinations were included in the analysis (i.e. pseudoviruses sensitive to N6, iMab, 10E8, CAP256-VRC26.25 and PG9). These included: 246-F3.C10.2, CNE8, CNE55, PVO4, X228, BJOX2000, CH119, 25710, CAP45, CE2017, CE1176, DU156, DU422 and ZM53 (n = 13). The Bliss-Hill Model in CombiNAber [29, 67] was used to predict IC_80_ values of the parental combination iMab + N6 against an expanded panel of 97 diverse pseudoviruses. Parental bNAbs (iMab and N6) IC_80_ and IC_50_ values used to generate Bliss-Hill predictions were sourced from the publicly available CATNAP database (http://hiv.lanl.gov/catnap) [68]. A total of 448 pseudoviruses were assessed from the CATNAP database but only 97 pseudoviruses with complete data (i.e., have both IC_80_ and IC_50_ data for both N6 and iMab) were used to generate Bliss-Hill combination predictions (the model requires complete data to generate predictions). The remaining 351 pseudoviruses were excluded.

### Statistical analysis

Comparisons of median neutralization IC_50_s and IC_80_s between iMab-N6 bibNAb and the parental antibodies (N6 or iMab) or parental antibody combination (iMab + N6) or additional bibNAbs (iMab-CAP256 or 10E8-iMab or PG9-iMab) were performed using a non-parametric, t-test (Wilcoxon matched-pairs signed rank test) and two-tailed *p* value. Where multiple comparisons between groups were performed, the level of significance was adjusted using Bonferroni correction (α/the number of comparisons; α = 0.05). *p* values were considered significant when the *p* value was < 0.01667 using the Bonferroni correction for 2 × 2 comparison of the iMab-N6 bibNAb to each parental antibody (iMab or N6) or the parental antibody combination (iMab + N6) or each bibNAbs (iMab-CAP256 or 10E8-iMab or PG9-iMab). Statistical analyses were performed using GraphPad Prism 7. The breadth-potency curves were generated by plotting the cumulative neutralization coverage against the experimentally determined or predicted IC_80_ values for each antibody using the survival model in GraphPad Prism 7.

## Results

### Antibody design and expression

iMab-N6 was designed using iMab-CAP256 [35] and 10E8-iMab [33] blueprints. The design incorporated CrossMab^CH1−CL^ and knob-in-a-hole mutations as described by Schaefer et al. [64] and Asokan et al. [63] respectively (left panel, Fig. 1A). These mutations allow for preferential assembly of the bibNAb, limiting unwanted by-products, without structurally compromising the paratope nor affecting epitope binding [63, 64]. iMab-N6 and its parental monoclonal antibodies were successfully expressed in HEK293T cells and purified using Protein-A agarose followed by Size-Exclusion Chromatography (SEC) (Fig. 1B). N6 chromatogram shows a homogenous sample whereas iMab-N6 and iMab (both produced using the pMin plasmid) chromatograms suggest a heterogenous sample (Fig. 1B). Elution fractions corresponding to the correctly assembled antibody confirmation were collected and pooled (shaded rectangles, Fig. 1B). The pooled SEC samples were resolved by a gradient SDS-PAGE under both reduced and non-reduced conditions to confirm purity and the correct assembly of the antibodies (Fig. 1C). Under SDS-PAGE, reduced conditions, both heavy chain and light chain bands resolved at the anticipated positions for all the antibodies suggesting correct assembly of the antibodies (Fig. 1C). Two distinct light chain bands were observed for the iMab-N6 bispecific antibody, each corresponding with the light chain bands of the parental monoclonal antibodies, iMab and N6 (Fig. 1C). Likewise, iMab-N6 heavy chains resolved into two distinct bands, each corresponding with the heavy chain bands of either iMab or N6 (Fig. 1C). Therefore, the heavy and light chain banding pattern observed for the purified iMab-N6 is consistent with parental monoclonal antibodies, suggesting correct assembly following in vitro expression. Under non-reduced conditions, the full antibodies resolved at a position between 135 and 190 kDa (Fig. 1C).

### Testing the functionality of the Fab moieties

**Fig. 2:**
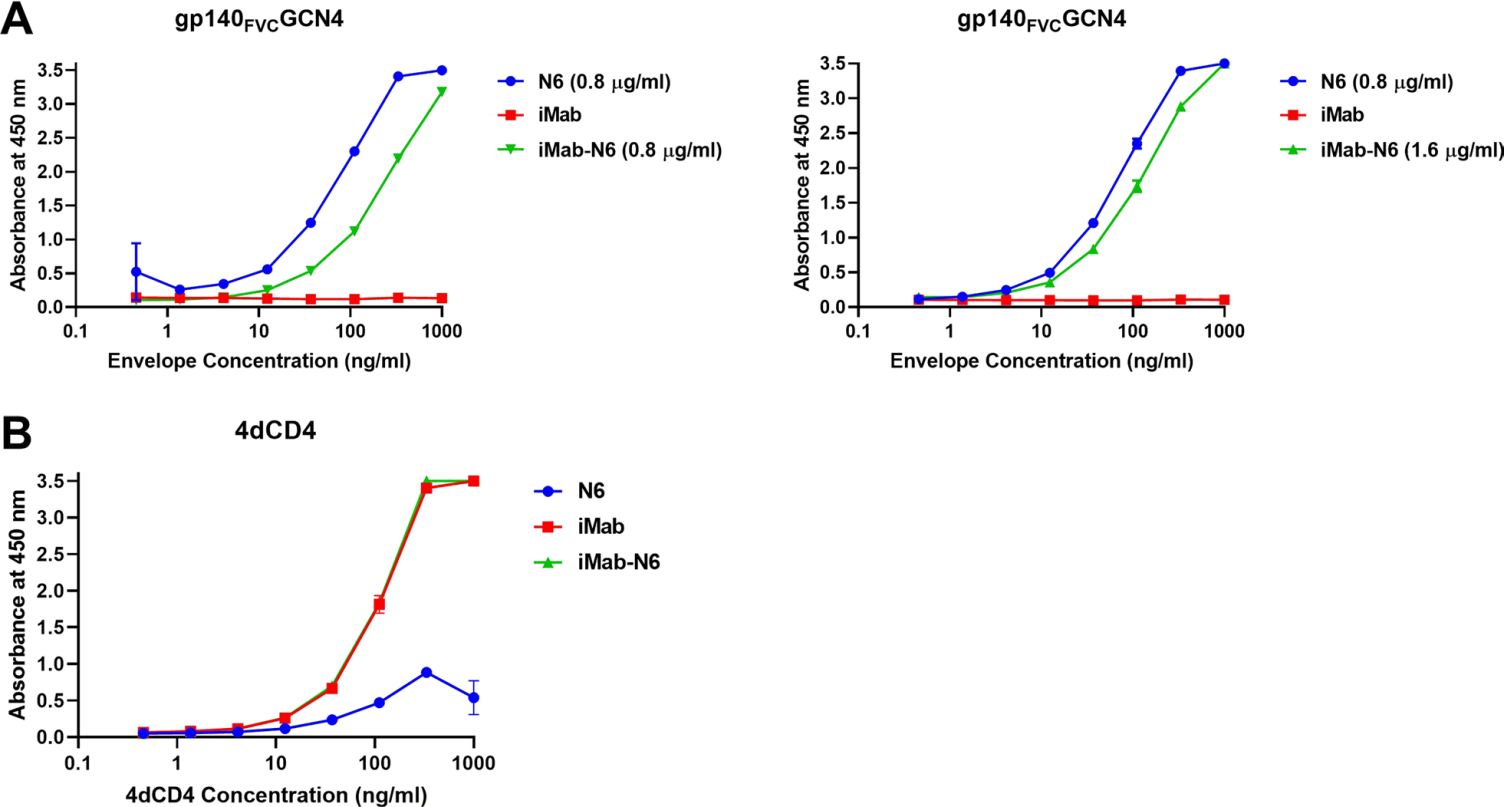
Binding characterisation of purified antibodies by ELISA. **A** Left panel: HIV-1 Env targeting moieties (N6 and iMab-N6) were tested against gp140_FVC_GCN4 with iMab being used as a negative control. **A** Right panel: iMab-N6 was used at double the concentration of N6 to try and correct for the difference in binding affinity seen in **A** Left panel. **B** CD4 binding moieties (iMab and iMab-N6) were tested against 4dCD4 with N6 serving as an appropriate negative control

Antibody binding capabilities and specificity were determined by means of an ELISA (Fig. 2). HIV-1 Env targeting moieties (N6 and iMab-**N6**) and the host-directed CD4 binding moieties (iMab and **iMab**-N6) were tested against gp140_FVC_GCN4 and 4dCD4, respectively. As expected, both N6 (blue trace) and iMab-**N6** (green trace) bound to HIV-1 Env gp140_FVC_GCN4 but iMab (red trace, CD4 targeting) did not, thus confirming the functionality of the HIV-1 Env targeting moieties (left panel, Fig. 2A). Interestingly, a reduction in the binding titres of iMab-N6 to gp140_FVC_GCN4 compared to N6 was evident (left panel, Fig. 2A). Doubling the concentration of iMab-N6 from 0.8 to 1.6 µg/ml to stoichiometrically match the number of N6 Fab fragments in the parental N6 monoclonal antibody (at 0.8 µg/ml) reduced the difference in the binding levels, although not completely (right panel, Fig. 2A). The CD4 binding moieties in iMab and **iMab**-N6 bound to 4dCD4 whilst N6 (HIV-1 Env targeting), did not. Notably, the binding levels of iMab and iMab-N6 to 4dCD4 are the same unlike N6 and iMab-N6 to gp140_FVC_GCN4 (Fig. 2). Here, we confirm the activity of both Fab moieties of the bispecific antibody.

### iMab-N6 displays enhanced breadth over parental constituents

**Fig. 3:**
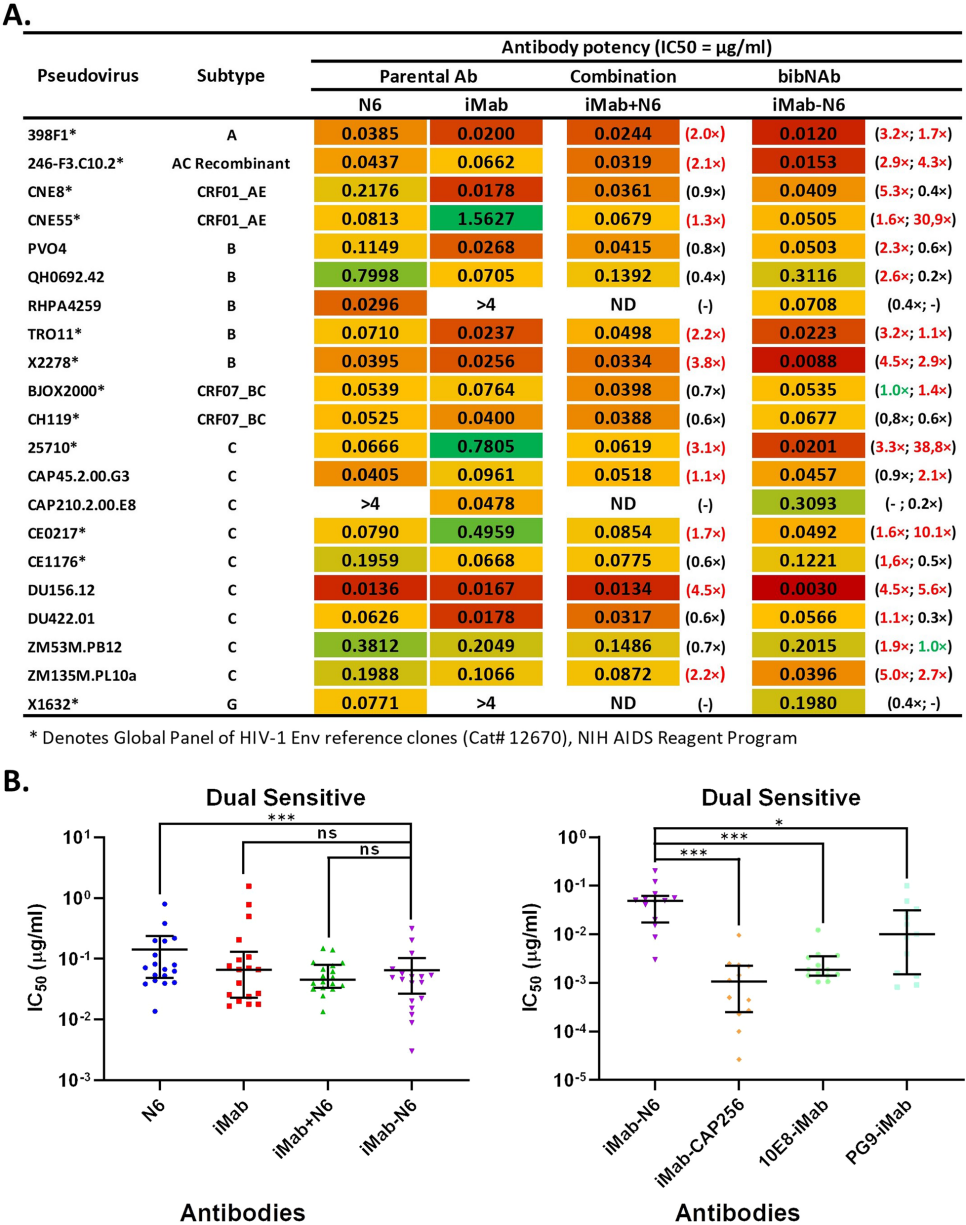
Comparison of antibody breadth and potency against a panel of 21 HIV-1 pseudoviruses. **A** IC_50_ heat map of the parental monoclonal antibodies alone, iMab and N6; antibody combination iMab + N6; and the bibNAb iMab-N6. The numbers in brackets next to iMab + N6 represent the fold difference in potency (red = enhancement, black = reduction) of iMab-N6 over iMab + N6. The numbers in brackets next to iMab-N6 represent the fold difference in potency (green = no enhancement) of iMab-N6 over parental N6 and iMab antibodies, respectively. Individual cells are colour-coded according to IC_50_ potency (number), with lower numbers and darker red representing greater potency. *ND* not done. **B** Scatter plot depicting IC_50_ values of dual sensitive HIV-1 pseudoviruses for each antibody with the median and IQR shown. Comparisons were done using non-parametric t-test (Wilcoxon matched-pairs signed rank test) with *p < 0.0167 and ***p < 0.001

Antibodies were tested against a panel of 21 geographically diverse HIV-1 pseudoviruses in a neutralisation assay to determine breadth and potency. The panel consists of both single sensitive (n = 3, CAP210.2.00.E8, RHPA4259 and X1632) and dual sensitive (n = 18) HIV-1 pseudoviruses. HIV-1 pseudoviruses from the global panel are part of the 21 selected HIV-1 pseudoviruses as they are representative of the globally circulating HIV-1 viruses [56]. When comparing iMab-N6 to its parental monoclonal antibodies, we observed an enhancement in the breadth of the bibNAb, neutralising 21/21 (100%) of the HIV-1 pseudoviruses compared to 20/21 (95%) by N6 and 19/21 (90%) by iMab (Fig. 3A and Additional file 1: Fig. S1). iMab-N6 showed no potency enhancement in comparison to iMab or N6 against the 3 single sensitive HIV-1 pseudoviruses (Fig. 3A and Additional file 1: Fig. S1). Examining the dual sensitive HIV-1 pseudoviruses, iMab-N6 in comparison to N6 exhibited a modest enhancement in potency (fold increase ranging from 1.1× to 5.3×) against 15/18 HIV-1 pseudoviruses, with no enhancement against BJOX2000 and a slight reduction in potency against CH119 and CAP45.2.00.G3 (fold reduction of 1.3× and 1.1× respectively) (Fig. 3A and Additional file 1: Fig. S1). An overall comparison of iMab-N6 to N6 showed a statistically significant enhancement in potency (*p* = 0.0001), however modest (Fig. 3B). Disappointingly, iMab-N6 did not exhibit an overall statistically significant enhancement in potency compared to iMab (*p* = 0.1674) (Fig. 3B). However, iMab-N6 did show noticeable enhancement in potency over iMab against 246-F3.C10.2, CNE55, 25,710, CE217 and DU156 (fold increase of 4.3×, 30.9×, 38.8×, 10.1× and 5.6× respectively) (Fig. 3A and Additional file 1: Fig. S1). Collectively, iMab-N6 only showed an enhancement in potency over both iMab and N6 against 9/18 dual sensitive HIV-1 pseudoviruses (fold increase ranging from 1.1× to 4.5×) (Fig. 3A and Additional file 1: Fig. S1). For completion, we compared iMab-N6 to a combination of the parental monoclonal antibodies (iMab + N6) against the 18 dual sensitive HIV-1 pseudoviruses. Both iMab-N6 and iMab + N6 were tested at a maximum concentration of 4 µg/ml with iMab + N6 prepared by combining 2 µg/ml of each parental monoclonal antibody. iMab-N6 exhibited an enhancement in potency over iMab + N6 against 10/18 dual sensitive HIV-1 pseudoviruses (fold increase ranging from 1.1× to 4. ×) (Fig. 3A and Additional file 2: Fig. S2). Of these ten, nine HIV-1 pseudoviruses (i.e., excluding CAP45.2.00.G3) are the same nine that iMab-N6 exhibited enhancement in potency over both iMab and N6. Unsurprising, an overall comparison of iMab-N6 to iMab + N6 showed no statistically significant difference (*p* = 0.1964) (Fig. 3B). Interested in exploring how iMab-N6 compares to other bibNAbs that have shown a statistically significant enhancement in neutralization over both their respective parental monoclonal antibodies, we compared IC_50_ values of iMab-N6 to iMab-CAP256 [35], 10E8-iMab [33] and PG9-iMab [34] against only those pseudoviruses that displayed sensitivity to all four parental antibodies. Disappointingly, all three bibNAbs showed statistically significant enhancement in potency over iMab-N6 (Fig. 3B). IC_50_ data for iMab-CAP256, 10E8-iMab and PG9-iMab were sourced from Moshoette et al.[35].

### Predicting the clinical potential of iMab-N6

**Fig. 4:**
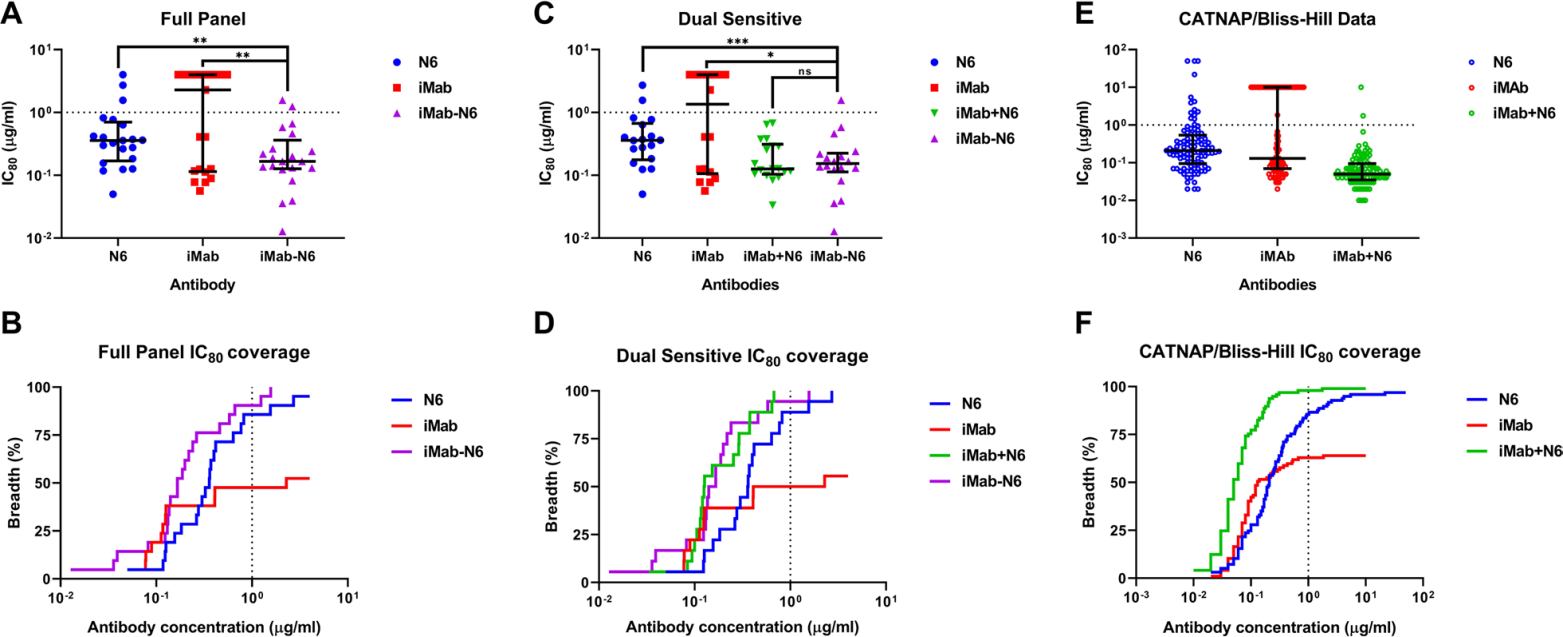
Predicting the clinical utility of iMab-N6. **A**–**F** Scatter plots depicting IC_80_ values with median and IQR shown (**A**, **C** and **E**), and Breadth-Potency curves (**B**, **D**, and **F**) of each antibody against the full panel (n = 21) (**A** and **B**), dual sensitive viruses (n = 18) (**C** and **D**) and the expanded CATNAP virus panel (n = 97) (**E** and **F**). **A**–**F** Dotted lines represent the IC_80_ = 1 µg/ml threshold. A and C Statistical analysis were done using non-parametric t-test (Wilcoxon matched-pairs signed rank test) with *p < 0.0167, **p < 0.005 and ***p < 0.001. **A**–**D** bibNAb iMab-N6 exhibited median IC_80_ 6–6.5× lower than 1 µg/ml AMP trial threshold and showed good neutralisation coverage of 90–94% at IC_80_ < 1 µg/ml. **E** and **F** N6 and iMab data was sourced from the CATNAP data base and used to generate iMab + N6, Bliss-Hill prediction data

In the AMP trial, VRC01 was 75% effective at preventing infection against viruses with an IC_80_ of < 1 µg/ml but offered no protection against those with an IC_80_ of > 1 µg/ml [18]. Based on this result, an in vitro IC_80_ cut-off of < 1 µg/ml is seen as a preliminary indicator for clinical PrEP efficacy. Accordingly, the full panel (n = 21) IC_80_ data of the parental bNAbs (N6 and iMab), and the bibNAb iMab-N6 were calculated and used for breadth and potency analysis (Fig. 4A, B). Interestingly, and in contrast to the IC_50_ data (Fig. 3B), iMab-N6 outperformed both parental bNAbs, N6 and iMab (p = 0.001, and *p* = 0.0043 respectively; median IC_80_ of 0.1664 µg/ml, 0.3582 µg/ml, and 2.289 µg/ml respectively) (Fig. 4A). Furthermore, iMab-N6 exhibited 100% IC_80_ coverage of the full panel in comparison to 95% by N6 (20/21) and a disappointing 52% by iMab (11/21) (Fig. 4A, B). Crucially, iMab-N6 showed better neutralisation coverage at IC_80_ < 1 µg/ml than both parental bNAbs, N6 and iMab [90% (19/21), 86% (18/21), and 48% (10/21) respectively] (Fig. 4A, B). Against the dual sensitive viruses (as defined by IC_50_ data in Fig. 3A), iMab-N6 showed no significant improvement compared to the parental combination iMab + N6, but outperformed both parental bNAbs N6 and iMab (*p* = 0.8, *p* = 0.0001, and *p* = 0.0077 respectively; median IC_80_ of 0.1534 µg/ml, 0.1263 µg/ml, 0.3601 µg/ml, and 1.349 µg/ml respectively) (Fig. 4C). The parental bNAb combination iMab + N6 achieved 100% (18/18) neutralisation coverage against the dual sensitive viruses at IC_80_ < 1 µg/ml, followed by iMab-N6 (94%, 17/18), N6 (89%, 16/18) and iMab (50%, 9/18) (Fig. 4C, D). Overall, iMab-N6 exhibits improved neutralisation coverage and potency over the parental bNAbs iMab and N6, but no significant difference against the iMab + N6 combination when assessing it against the IC_80_ < 1 µg/ml criteria. Given the similar neutralisation profile of iMab-N6 and iMab + N6, the Bliss-Hill model [29] was used to predict breadth and potency data of the combination against an expanded HIV-1 panel of 97 diverse pseudoviruses (Fig. 4E, F). This data serves as an indirect indicator of the bibNAb iMab-N6 performance against an expanded panel of pseudoviruses, pending actual in vitro testing. Consistent with our own data, the Bliss-Hill model predicted a median IC_80_ lower than 1 µg/ml for the combination (0.05 µg/ml) with a 98% (95/97) neutralisation coverage at IC_80_ < 1 µg/ml (Fig. 4E, F). The parental CATNAP data plotted alongside the Bliss-Hill model predictions also produced median IC_80_ values of less than 1 µg/ml for the parental bNAbs (N6: 0.21 µg/ml and iMab: 0.13 µg/ml) with a neutralisation coverage at IC_80_ < 1 µg/ml of 86% (83/97) and 63% (61/97) for N6 and iMab, respectively (Fig. 4E, F). Overall, the iMab + N6 Bliss-Hill combination data suggests that iMab-N6 would perform well against an expanded panel of pseudoviruses and predicts superior clinical performance compared to VRC01 based on the available IC_80_ data.

## Discussion

The isolation of second generation bNAbs with exceptional neutralisation coverage and/or potency has reinvigorated the HIV-1 bNAb PrEP/therapeutic field as many potential clinical candidates have been identified. Moreover, the recently concluded phase IIb clinical trial (AMP Trial: HVTN 703/HPTN 081 and HVTN704/HPTN 085) has validated bNAbs as potential PrEP candidates subject to a selection that maximises neutralising breadth and potency of the circulating regional/global HIV-1 strains. However, pre-existing, and new resistance mutations has necessitated the use of bNAb combinations for antibody PrEP/therapy strategies. Here we report on the engineering of a bibNAb using a host CD4 targeting antibody, iMab [48] paired with an HIV-1 Env CD4bs targeting bNAb, N6 [7]. By combining iMab (92% breadth with a median IC_50_ of 0.03 µg/ml against diverse HIV-1 Env subtypes) with N6 (98% breadth with a median IC_50_ of 0.066 µg/ml against HIV-1 subtype C), we hoped to engineer a bibNAb with exceptional breadth and potency against HIV-1 subtype C, the predominant subtype globally [7, 47, 48]. Like iMab-CAP256 [35] and 10E8-iMab [33], iMab-N6 was designed using the normal antibody architecture and with the addition of Knob-in-a-hole and CrossMab^CH1−CL^ mutations (Fig. 1A). These mutations promote the correct assembly of the bibNAb and do not interfere with its function [63, 64].

Having successfully produced and purified iMab-N6 (Fig. 1), we assessed the binding of the Fab moieties to their respective epitopes (Fig. 2). iMab-N6 bound to both gp140_FVC_GCN4 and 4dCD4 thereby confirming the functionality of both the Fab moieties. Interestingly, iMab-N6 showed lower binding levels to gp140_FVC_GCN4 in comparison to N6, this difference was reduced when the concentration of iMab-N6 was doubled within the assay (Fig. 2A). Whilst the ELISA methodology may not provide sufficient quantitative measure of binding kinetics of the parental N6 compared to the bispecific antibody, these data taken together suggest that the parental N6 antibody displays more favourable binding kinetics. Whether this was due to difference in avidity and/or the bivalent nature of the N6 parental or the immobilized Env glycoprotein that promoted dual engagement remains unknown. Nonetheless, this data does confirm the functionality of both N6 and iMab Fab moieties of the bispecific, albeit with a perhaps reduced Env-affinity.

Having confirmed the functionality of the Fab moieties, we next determined the breadth and potency of iMab-N6 against a panel of 21 (inclusive of the global panel) geographically diverse HIV-1 pseudoviruses (Fig. 3). The selected panel consists of both single sensitive (n = 3, CAP210.2.00.E8, RHPA4259 and X1632) and dual sensitive (n = 18) HIV-1 pseudoviruses. iMab-N6 exhibited an enhancement in neutralisation breadth over its two parental bNAbs; neutralising 21/21 of the HIV-1 pseudoviruses compared to 20/21 by N6 and 19/21 by iMab (Fig. 3A). This improvement in the neutralization breadth was anticipated given the incorporation of only single resistant viral strains within the assay, and once again confirms the independent, functionality of both Fab moieties of the iMb-N6 bispecific conformation. Against the dual sensitive pseudoviruses, iMab-N6 exhibited a modest, yet statistically significant enhancement in potency in comparison to N6 but not iMab (*p* = 0.0001 and *p* = 0.1674 respectively, median IC_50_ of 0.0475 µg/ml, 0.0688 µg/ml, and 0.0665 µg/ml respectively) (Fig. 3B). iMab-N6 did show noticeable enhancement in potency over iMab against 246-F3.C10.2, CNE55, 25710, CE217 and DU156 (fold increase of 4.3×, 30.9×, 38.8×, 10.1× and 5.6×, respectively) (Fig. 3A). However, this enhancement in potency can be solely attributed to the improved potency of N6 against these isolates compared to the parental iMab. Consistent with the above findings, no enhancement in potency was noted in relation to the parental bNAb combination iMab + N6 (*p* = 0.1964, median IC_50_: combination 0.0457 µg/ml vs. bibNAb 0.0475 µg/ml) (Fig. 3B). Taken together, these data suggest a lack of synergy (i.e., simultaneous binding to both epitopes) between the bibNAb Fab moieties, albeit in respect to the current bispecific conformation under evaluation. The simultaneous engagement of both epitopes, improves the avidity of these bispecifics which may translate into increased potency [33, 34, 69]. Additionally, for iMab-based bibNAbs, engagement of the host CD4 by the iMab Fab moiety concentrates the bibNAb at the site of viral entry, enabling the other Fab moiety to engage HIV-1 Env thereby neutralising the virus [33, 34]. Potency enhancement of bibNAbs is not guaranteed as has been observed previously for other bibNabs reported in the literature to date; e.g. 3BNC117-PGT135 WT [69], PGT128-iMab [33] and 3BNC117-iMab [33]. Of particular interest, the latter, 3BNC117-iMab shares structural (Cross Mab/KIH assembly) and antigen-targeting (CD4bs HIV-1 spike/domain 2 of host CD4) similarities to the iMab-N6 bispecific described herein, also did not convey significant potency enhancement compared to the parental antibodies [33]. Whether this statement would hold for the expanded class of CD4bs-directed antibodies in combination with iMab, or that further improvements in neutralization potency could be achieved through structural optimization remain to be determined. For example; Bournazos et al. [69] improved the potency of 3BNC117-PGT135 WT by introducing the flexible hinge domain of IgG3 to the bibNAb while retaining the IgG1-Fc region. The increased flexibility allowed 3BNC117-PGT135 to simultaneously engage both its epitopes, thereby enhancing its potency [more potent than WT 3BNC117-PGT135 and the parental bNAbs (3BNC117 and PGT135)] [69]. Alternatively, iMab-N6 potency may be improved by completely switching IgG1 for IgG3 (class switching) instead of only introducing the hinge region to an IgG1 structure. Richardson et al. [70] showed that CAP256 (highly potent bNAb) potency was significantly improved by an IgG1 to IgG3 class switch. Like with 3BNC117-PGT135, the enhancement in potency was attributed to increased flexibility which translates to better epitope accessibility and affinity [70]. Class switching has the added benefit of IgG3 Fc effector functions which are highly desirable in HIV-1 treatment and prevention strategies [70, 71]. In addition, the neutralisation breadth of iMab-N6 may be bolstered further by replacing wild type (WT) iMab with modified iMab LM52 [72] in the bibNAb configuration. Song et al. [72] engineered iMab LM52 by introducing an N-linked glycan to the iMab light chain thereby overcoming iMab resistant pseudoviruses. This single modification to iMab improved its neutralisation breadth from 92% coverage to 100% coverage of the 118 pseudoviruses tested [72]. Combined, these strategies highlight the complexity when optimizing for bispecific conformations for improved potency and breadth against circulating HIV-1.

Recently, the results AMP clinical trials designed as proof-of-concept for bNAbs prophylaxis against HIV-1 acquisition were reported. Overall, VRC01 failed to protect against HIV-1 acquisition relative to the placebo control group. However, VRC01 conferred 75% protective efficacy against VRC01-sensitive viruses (IC_80_ < 1 µg/ml), accounting for 30% of the viruses circulating within the trial regions compared to the placebo control group. The AMP trial findings highlight the role of neutralization breadth and potency in ultimately determining clinical utility. The trial data does suggest that an in vitro, IC_80_ cut off value < 1 µg/ml may hold real-world application in predicting the success of said strategies. Given the lack of enhancement in potency of iMab-N6 against its parental bNAbs, it came as no surprise that iMab-N6 is the lowest performing bibNAb in comparison to iMab-CAP256 [35], 10E8-iMab [33] (the two bibNAbs that share the same Ab structural architecture as iMab-N6) and PG9-iMab [34] (Fig. 3B). However, we were encouraged to note that that the median IC_80_ data generated (against the dual sensitive pseudoviruses) here was approximately 2.8–7.9-fold lower than the 1 µg/ml AMP trial threshold, for the N6 parental, parental combination and the bispecific (N6 0.358 µg/ml, iMab 2.29 µg/ml, iMab + N6 0.126 µg/ml and iMab-N6 0.166 µg/ml) (Fig. 4A, C). Moreover, iMab-N6 has a 90% IC_80_ coverage at 1 µg/ml in comparison to 86% for N6 and 48% for iMab against the full panel; indicating a significant improvement over the parental bNAbs (Fig. 4A, B). While confirmation of the iMab-N6 bibNAb median neutralisation potency would require validation against a more comprehensive pseudovirus panel, Bliss-Hill [29] prediction of the iMab + N6 combination suggests an IC_80_ < 1 µg/ml would be achievable for the bibNAb iMab-N6 (Bliss-Hill prediction iMab + N6: median IC_80_ of 0.05 µg/ml and a 98% (95/97) neutralisation coverage at IC_80_ < 1 µg/ml) (Fig. 4E, F). In conclusion, bibNAb iMab-N6 engineered using the normal Ab architecture with the addition of CrossMab^CH1−CL^ and Knob-in-a-hole mutations, exhibits enhancement in breadth but not potency against its parental bNAbs. Moreover, no enhancement in potency was observed in comparison to the parental bNAb combination suggesting a lack of synergy between the two iMab-N6 Fab moieties. We believe the re-engineering iMab-N6 to further enhance its potency using the strategies described above is a worthwhile endeavour due to the need for a large war chest in the fight against HIV-1.

## Supplementary Information

**Additional file 1: Fig. S1.** Neutralisation curves showing breadth of iMab-N6 in comparison to the parental antibodies iMab and N6. iMab-N6 displayed an improvement in breadth by neutralising 21/21 of the HIV-1 pseudovirus compared to 20/21 by N6 and 19/21 by iMab. iMab-N6 showed a reduction in potency (as shown by the right shift of the bibNAb line) over the active parental monoclonal antibody against the three single-sensitive HIV-1 pseudoviruses (RHPA4259.7, X1632 and CAP210.2.00E8). iMab-N6 exhibited enhancement in potency (as shown by the left shift of the bibNAb line) over N6 and iMab against 15/18 and 11/18 dual sensitive HIV-1 pseudoviruses. Collectively, iMab-N6 only showed an enhancement over both iMab and N6 against 9/18 dual sensitive HIV-1 pseudoviruses (fold increase ranging from 1.1× to 4.5×). iMab, N6 and iMab-N6 were tested at a maximum concentration of 4 µg/ml.

**Additional file 2: Fig. S2.** Neutralisation curves of iMab-N6 in comparison to the parental combination (iMab + N6). iMab-N6 exhibited an enhancement in potency over iMab + N6 against 10/18 dual sensitive HIV-1 pseudoviruses (fold increase ranging from 1.1× to 4.5×). Of these ten, nine HIV-1 pseudoviruses (i.e., excluding CAP45.2.00.G3) are the same nine that iMab-N6 exhibited enhancement in potency over both iMab and N6. Results suggest N6/iMab bispecific structural configuration of these paratopes are crucial for the observed enhancement in potency against these 9 HIV-1 pseudoviruses. Both iMab-N6 and iMab + N6 were tested at a maximum concentration of 4 µg/ml with the iMab + N6 combination prepared by mixing 2 µg/ml of each parental monoclonal antibody.

## Data Availability

The datasets used and/or analysed during the current study are available from the corresponding author upon request. Parental bNAbs (iMab and N6) IC_80_ and IC_50_ values used to generate Bliss-Hill predictions were sourced from the publicly available CATNAP database (http://hiv.lanl.gov/catnap).

## Abbreviations

ART: Antiretroviral therapy
bNAbs: Broadly neutralising antibodies
bibNAbs: Bispecific bNAbs
tribNAbs: Trispecific bNAbs
Env: HIV-1 envelope glycoprotein
Fab: Fragment antigen-binding
FDA: Food and Drug Administration
HEK293T cells: Human embryonic kidney 293 T antigen cells
IC_50_ or IC_80_: 50% Or 80% inhibitory concentration
iMab: Ibalizumab
iMab-N6: Ibalizumab-N6
iMab-CAP256: Ibalizumab-CAP256.VRC26.25
10E8-iMab: 10E8-ibalizumab
PG9-iMab: PG9-ibalizumab
pMin: MAb expression vector
SDSPAGE: Sodium dodecyl sulphate polyacrylamide gel electrophoresis
SEC: Size exclusion chromatography
TCID_50_: 50% tissue culture infectious dose
ELISA: Enzyme-linked immunosorbent assay
